# How to isolate the superior vena cava using balloon-in-basket pulsed field ablation system: Standardized workflow and initial results

**DOI:** 10.1016/j.hroo.2026.01.014

**Published:** 2026-01-17

**Authors:** Roland Richard Tilz, Kohei Ukita, Charlotte Eitel, Karl-Heinz Kuck, Sascha Hatahet, Jan-Per Wenzel

**Affiliations:** 1 Department of Rhythmology, University Heart Center Lübeck, University Hospital Schleswig-Holstein, Lübeck, Germany; 2 German Center for Cardiovascular Research, Partner Site Lübeck, Germany

**Keywords:** Atrial fibrillation, Catheter ablation, Pulmonary vein isolation, Pulsed field ablation, Superior vena cava

## Abstract

**Background:**

Superior vena cava (SVC) is one of the most important non–pulmonary vein (PV) foci in patients with atrial fibrillation (AF), but ablation at this site carries risks of phrenic nerve injury and sinus node dysfunction. Balloon-in-basket (BiB)-pulsed field ablation (PFA) is a novel nonthermal system offering stable wall contact and simultaneous mapping, yet its use for SVC isolation has not been well established.

**Objective:**

This study described a standardized workflow and initial results of SVC isolation using the BiB-PFA system.

**Methods:**

We included patients who underwent de novo PV isolation and SVC isolation using the BiB-PFA system. SVC isolation was performed using a standardized workflow including electroanatomic mapping, phrenic nerve pacing, selective voltage adjustment (1800 or 1400 V), and systematic remapping. Baseline characteristics, procedural details, and acute safety outcomes were evaluated.

**Results:**

A total of 10 patients (median age 78 years, 4 women, and 2 with paroxysmal AF) were analyzed. Acute PV isolation and SVC isolation were achieved in all patients. 1 patient developed transient sinus node arrest immediately after the first application for SVC, which resolved with isoprenaline without pacemaker implantation. No persistent sinus node dysfunction or phrenic nerve palsy occurred.

**Conclusion:**

We described a standardized, reproducible strategy for SVC isolation using the BiB-PFA system. In this initial experience, the strategy was feasible and safe, with acute success in all patients and only 1 transient adverse event. These findings suggest that the BiB-PFA system may represent a promising tool for adjunctive SVC ablation in AF.

## Graphical abstract

**Figure:**
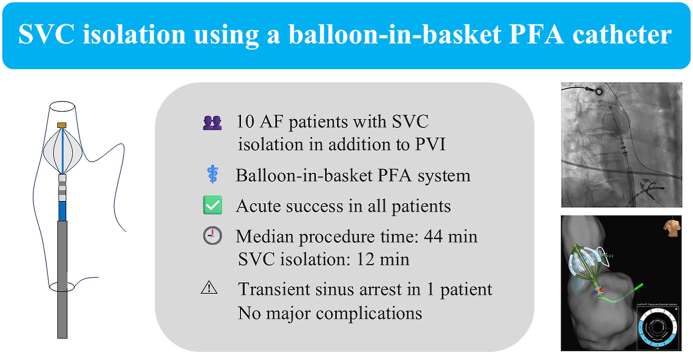

**Key Findings**

▪ A standardized workflow using the balloon-in-basket pulsed field ablation (BiB-PFA) system enabled acute superior vena cava (SVC) isolation in all patients undergoing de novo atrial fibrillation ablation.
▪ The BiB-PFA–based SVC isolation strategy showed a favorable acute safety profile, with no phrenic nerve palsy or major procedural complications; 1 transient sinus node arrest resolved without sequelae.
▪ Stable balloon-based wall contact and simultaneous mapping allowed efficient and reproducible SVC isolation with a limited number of applications and short procedural time, supporting the feasibility of this approach.

## Introduction

The superior vena cava (SVC) has been recognized as one of the most important non–pulmonary vein (PV) foci contributing to the initiation and maintenance of atrial fibrillation (AF).^1^, ^2^, ^3^ However, SVC isolation with thermal energies (radiofrequency or cryoablation) has been associated with procedural complications including phrenic nerve injury and sinus node dysfunction.^4^^,^^5^

Pulsed field ablation (PFA) has recently emerged as a nonthermal modality based on irreversible electroporation, allowing for myocardial tissue selectivity while reducing the risk of collateral damage.^6^ Previous reports using PFA catheters demonstrated the feasibility of SVC isolation and suggested a favorable safety profile compared with thermal energies.^7^, ^8^, ^9^

The balloon-in-basket (BiB)-PFA is a novel system that combines a compliant balloon with an expandable multielectrode lattice structure, designed to ensure stable catheter positioning and consistent electrode–tissue contact.^10^, ^11^, ^12^ However, little has been reported on the use of the BiB-PFA system for SVC isolation.

In this report, we described a standardized procedural workflow in SVC isolation using the BiB-PFA system and present baseline characteristics, procedural outcomes, and acute safety data from the first 10 consecutive cases performed at a high-volume center.

## Methods

### Study population

We included consecutive patients undergoing both PV and SVC isolation using the BiB-PFA system (VOLT, Abbott, St. Paul, MN) as an initial catheter ablation for AF between June 2025 and December 2025 at the Heart Center Lübeck. Patient characteristics, procedural details, and procedure-related complications were analyzed.

The study protocol was approved by the local ethics committee (Lübeck Ablation Registry, approval number WF-028/15) and conducted in accordance with the Declaration of Helsinki. A written informed consent for catheter ablation and participation in the study was obtained from all patients.

### General procedural management

All patients underwent a standardized preprocedural workup in line with institutional protocols. In patients with an elevated thromboembolic risk, transesophageal echocardiography was performed to exclude the presence of intracardiac thrombi. Anticoagulation management followed institutional protocols: vitamin K antagonists were continued within the therapeutic international normalized ratio range (2.0–3.0), whereas direct oral anticoagulants were withheld on the morning of the procedure.

Ablation procedures were performed under either deep sedation with propofol or light sedation without propofol (midazolam, fentanyl, metamizole, and lidocaine) at the discretion of the treating electrophysiologist. Vascular access was obtained via 2 ultrasound-guided punctures of the femoral vein using 8F sheaths. A diagnostic catheter was placed in the coronary sinus. Transseptal puncture was performed with a modified Brockenbrough technique under fluoroscopic guidance, and unfractionated heparin was administered to maintain an activated clotting time of >300 seconds. A steerable 13F sheath (Agilis NxT, Abbott) was used to introduce the BiB-PFA catheter into the left atrium (LA). The 3-dimensional geometry of the LA and PVs was reconstructed using a mapping system (EnSite X, Abbott). PV isolation was performed with at least 2 rotated applications per PV at nominal voltage (1800 V). If phrenic nerve capture was detected in right-sided PVs, energy delivery was reduced to 1400 V with a minimum of 3 applications. A maximum of 8 applications per PV was permitted. Acute isolation was confirmed by remapping with the BiB-PFA catheter.

In addition, LA posterior wall (LAPW) isolation was performed using the BiB-PFA catheter in patients with low-voltage areas in the LAPW. For LAPW ablation, the BiB-PFA catheter was positioned against the LAPW and splines facing the LA anterior wall were deselected. Acute isolation of the LAPW was confirmed by remapping with the BiB-PFA catheter.

### SVC isolation

All patients in this study underwent SVC isolation. The indication for SVC isolation was AF of SVC origin or frequent premature atrial contractions arising from the SVC. In patients with AF rhythm prior to SVC isolation, sinus rhythm was restored by cardioversion. An activation map of the right atrium (RA) and SVC was obtained using the BiB-PFA catheter. The earliest activation site was identified to localize the sinus node region, which was carefully avoided during ablation. A 0.035-in guidewire was advanced through the BiB-PFA catheter into the SVC, and contrast injection delineated the SVC–RA junction. The BiB-PFA catheter was then positioned at the SVC–RA junction, inflated with 8–10 mL until SVC wall contact was achieved, and gently advanced to ensure circumferential contact slightly superior to the sinus node region. Phrenic nerve integrity was assessed by high-output pacing. Energy was delivered at 1800 V. If phrenic nerve capture was present, voltage was reduced to 1400 V. A single test application (1 train of pulses) was performed to assess for sinus node dysfunction. In the event of bradycardia or asystole, the catheter was repositioned and advanced 3 mm deeper into the SVC. If no adverse response occurred, 2–4 applications were delivered: 1 circumferential application (10 trains) followed by a slight rotation of the catheter and the after application to address interspline gaps. After ablation, remapping with the BiB-PFA catheter was systematically performed to confirm complete SVC isolation.

### Postprocedural management

Hemostasis was achieved using vascular closure devices or figure-of-8 sutures combined with a compression bandage. The bandage was removed 4 hours after the end of the ablation procedure, and the suture was removed 4 hours after the bandage removal. Transthoracic echocardiography was performed immediately after the procedure, at 1 hour, and on the first postprocedural day to exclude pericardial effusion. The patients were discharged on the second postprocedural day.

### Statistical analysis

Categorical variables are reported as absolute and relative frequencies. Continuous data are presented as medians (interquartile ranges). All statistical analyses were performed using JMP 17 software (SAS Institute).

## Results

### Baseline characteristics

The baseline characteristics of the patients are presented in Table 1. The median age was 78 years, 4 patients (40%) were female, 2 patients (20%) had paroxysmal AF, and the median CHA_2_DS_2_-VA score was 3. The median LA volume index was 38 mL/m^2^, and the median left ventricular ejection fraction was 55%.

**Table 1:** Baseline characteristics of the patients

Variable	Total (n = 10)
Age (y)	78 (73–81)
Sex (female), n (%)	4 (40)
BMI (kg/m^2^)	27 (22–28)
Type of AF	
Paroxysmal AF, n (%)	2 (20)
Persistent AF, n (%)	8 (80)
Arterial hypertension, n (%)	7 (70)
Diabetes mellitus, n (%)	3 (30)
Congestive heart failure, n (%)	1 (10)
CHA_2_DS_2_-VA score	3 (2–4)
LAVI (mL/m^2^)	38 (33–48)
LVEF (%)	55 (53–60)
NT-proBNP (ng/L)	754 (285–1897)

AF = atrial fibrillation; BMI = body mass index; LAVI = left atrial volume index; LVEF = left ventricular ejection fraction; NT-proBNP = N-terminal pro-brain natriuretic peptide.

### Procedural characteristics

Procedural characteristics are presented in Table 2. All patients underwent successful PV isolation using the BiB-PFA catheter. LAPW isolation was performed in 9 patients (90%).

**Table 2:** Procedural characteristics

Variable	Total (n = 10)
Procedure time (min)	44 (40–67)
Fluoroscopy time (min)	7 (5–10)
Dose area product (cGy·cm^2^)	269 (209–806)
PVI, n (%)	10 (100)
Number of applications for PV	14 (10–16)
SVC isolation, n (%)	10 (100)
Number of applications for SVC	3 (2–4)
Duration of SVC isolation (min)	12 (9–15)
LAPW isolation, n (%)	9 (90)
Number of applications for LAPW	5 (4–6)

LAPW = left atrial posterior wall; PV = pulmonary vein; PVI = pulmonary vein isolation; SVC = superior vena cava.

**Figure 1:**
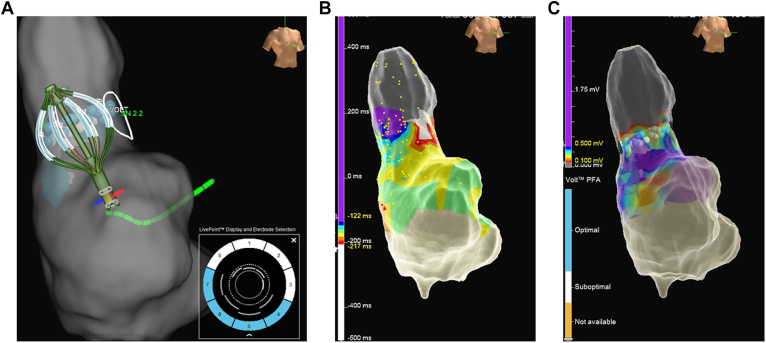
3-dimensional mappings during SVC isolation. **A:** A 3-dimensional map when applications were delivered using the BiB-PFA catheter (30-degree RAO view). **B:** An activation map of the RA and SVC obtained with the BiB-PFA catheter (30-degree RAO view). Sinus node region was identified as the *white area*. **C:** A voltage map obtained with the BiB-PFA catheter after SVC isolation (30-degree RAO view). BiB = balloon-in-basket; PFA = pulsed field ablation; RA = right atrium; RAO = right anterior oblique; SVC = superior vena cava.

**Figure 2:**
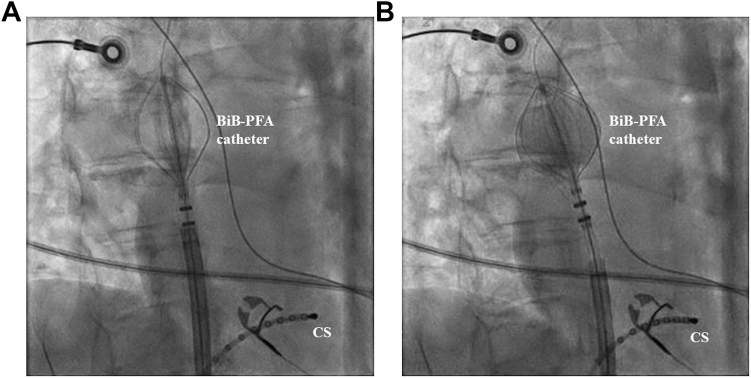
Fluoroscopic images during SVC isolation. **A:** A fluoroscopic image when the BiB-PFA catheter was positioned at the SVC–RA junction (30-degree RAO view). **B:** A fluoroscopic image when the BiB-PFA catheter was inflated with 8–10 mL until SVC wall contact was achieved (30-degree RAO view). BiB = balloon-in-basket; CS = coronary sinus; PFA = pulsed field ablation; RA = right atrium; RAO = right anterior oblique; SVC = superior vena cava.

**Figure 3:**
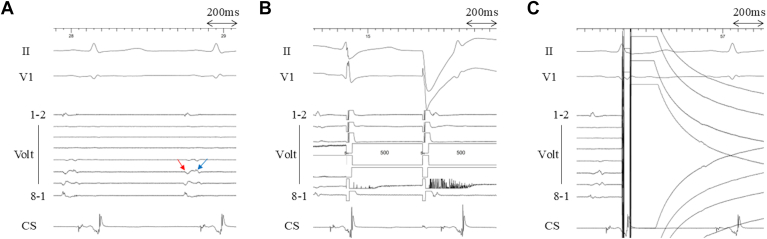
Intracardiac electrograms during SVC isolation. **A:** An intracardiac electrogram when the BiB-PFA catheter was inflated. The RA potentials (*red arrow*) and the SVC potentials (*blue arrow*) were observed. **B:** An intracardiac electrogram when the phrenic nerve integrity was assessed by high-output pacing from the BiB-PFA catheter. **C:** An intracardiac electrocardiogram when applications were delivered using the BiB-PFA catheter. BiB = balloon-in-basket; CS = coronary sinus; PFA = pulsed field ablation; RA = right atrium; RAO = right anterior oblique; SVC = superior vena cava.

SVC isolation was attempted in all patients and was acutely successful in 100% (10 of 10), requiring a median of 3 applications (2–4) per patient. The workflow in a representative case is shown in Figure 1, Figure 2, Figure 3. The position of the BiB-PFA catheter during applications is shown in Figure 1A, and the preablation activation map and the postablation voltage map are shown in Figure 1B and 1C, respectively. The fluoroscopic images are shown in Figure 2. After the BiB-PFA catheter was positioned at the SVC–RA junction (Figure 2A), the balloon was inflated with 8–10 mL until SVC wall contact was achieved (Figure 2B). The intracardiac electrograms are shown in Figure 3A. After RA and SVC potentials were identified (Figure 3A) and phrenic nerve integrity was assessed by high-output pacing (Figure 3B), a single test application (1 train of pulses) was performed to assess for sinus node dysfunction and subsequently applications were delivered (Figure 3C).

The median procedure time was 44 minutes, and the median time for SVC isolation was 12 minutes.

### Procedure-related complications

**Figure 4:**
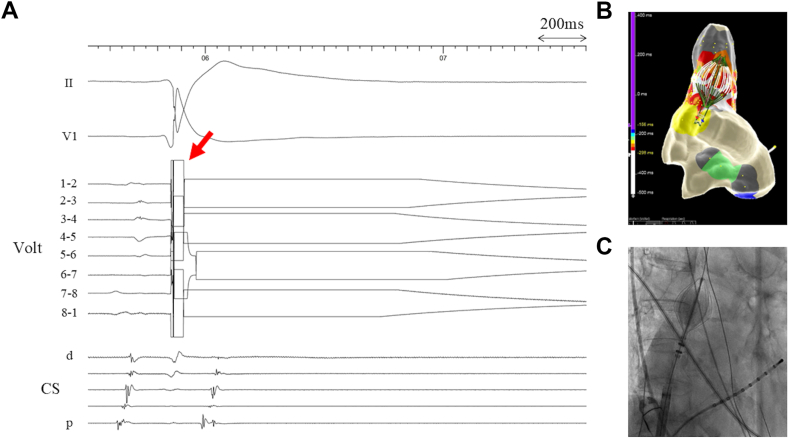
A case where a transient sinus node arrest was observed after the application. **A:** An intracardiac electrogram showing transient sinus node arrest after the application. The *red arrow* indicates the first PFA application at 1800 V. Sinus arrest occurred immediately after energy delivery. Sinus rhythm restored 30 seconds after the sinus arrest under isoprenaline infusion. **B:** An activation map showing the SVC–RA junction and the position of the BiB-PFA catheter (30-degree RAO view). The *white area* indicates the sinus node. **C:** A fluoroscopic image during the application using the BiB-PFA catheter (30-degree RAO view). BiB = balloon-in-basket; CS = coronary sinus; PFA = pulsed field ablation; RA = right atrium; SVC = superior vena cava.

The procedure-related complications are presented in Table 3. 1 patient experienced a transient sinus node arrest immediately after the application for SVC (Figure 4). Transient atrial stimulation via a multipolar catheter positioned in the coronary sinus was performed. Subsequently, intravenous isoprenaline was administered (4 μg bolus and 2 μg/min by intravenous infusion). Sinus rhythm recovered and stabilized 30 seconds after the sinus arrest. The infusion was discontinued after 10 minutes, after which the patient maintained a stable and persistent sinus rhythm (65 beats per minute). Sheaths were removed, and the patient was transferred to a regular monitored ward for 2 nights before being discharged in stable sinus rhythm.

**Table 3:** Procedure-related complications

Variable	Total (n = 10)
Cardiac tamponade, n (%)	0 (0)
Stroke, n (%)	0 (0)
Femoral pseudoaneurysm, n (%)	0 (0)
Phrenic nerve injury, n (%)	0 (0)
Bradycardia requiring transient pacing, n (%)	1 (10)
Bradycardia requiring permanent pacing, n (%)	0 (0)

No other arrhythmic complications were observed. No patient developed pericardial effusion, phrenic nerve palsy, stroke, groin complications, or vascular injury. No major adverse events occurred during hospitalization.

## Discussion

### Main findings

This study investigated the feasibility and safety of SVC isolation using the BiB-PFA system. The main findings are as follows:

1. SVC isolation using the BiB-PFA catheter was feasible and acutely successful in all 10 patients.
2. The workflow was reproducible and safe; only 1 transient episode of sinus node arrest occurred, which resolved without sequelae.
3. No major complications occurred, including phrenic nerve palsy, pericardial effusion, stroke, or vascular injury.

### Feasibility

Our findings demonstrate that SVC isolation with the BiB-PFA system can be consistently achieved using a standardized stepwise workflow. Previous studies with multipolar or pentaspline PFA catheters have shown similarly high acute success rates for SVC isolation, highlighting the potential of nonthermal energy at this site.^7^^,^^8^ However, these reports also raised concerns regarding phrenic nerve injury and sinus node dysfunction.

The workflow described in the present study was developed through an iterative process during our initial clinical experience with the BiB-PFA system. Early cases highlighted the importance of several procedural steps to ensure both efficacy and safety. In particular, systematic activation mapping of the RA and SVC was incorporated to accurately identify the sinus node region, which was then deliberately avoided during energy delivery. In addition, routine phrenic nerve pacing before ablation and selective voltage adjustment were adopted to minimize the risk of collateral injury. Importantly, the introduction of a single preliminary “test” PFA application before completing SVC isolation emerged as a key safety measure to assess susceptibility to sinus node dysfunction and guided catheter repositioning when necessary. These refinements were progressively integrated into a standardized and reproducible workflow.

The favorable feasibility profile of the BiB-PFA system is attributable to its catheter design and electrode configuration. The semicompliant balloon provides stable circumferential wall contact and naturally conforms to the SVC–RA junction, enabling uniform electrode–tissue contact. The flat electrode splines are arranged around the balloon periphery and oriented laterally, directing the electric field into the adjacent myocardium while minimizing energy dispersion into the blood pool.^11^

Compared with other PFA platforms currently used for SVC isolation, such as pentaspline or circular multielectrode array catheters, the BiB-PFA system offers several distinct procedural advantages. The balloon-based design facilitates stable circumferential contact at the SVC–RA junction without the need for complex catheter manipulation, whereas the lateral orientation of the splines directs energy preferentially toward the myocardial wall. In addition, the ability to perform simultaneous mapping and ablation with the same catheter allows real-time assessment of local electrograms and immediate confirmation of isolation, reducing the need for catheter exchange or repeated repositioning. These design characteristics directly influenced the development of the present workflow and differentiate it from strategies used with other PFA technologies.

Although a case of successful SVC isolation using the BiB-PFA catheter has already been reported, to the best of our knowledge, no data have been published involving a larger series of cases.^13^ In this series, SVC isolation was consistently achieved with the first application and a median of 3 applications, each application consisting of 10 PFA trains per patient, reflecting the high per-application efficacy of this system at the SVC–RA junction.

### Safety considerations

Application at the SVC is inherently associated with unique safety challenges, particularly the risk of phrenic nerve injury and sinus node dysfunction. Conventional thermal energy has been limited by these concerns, with phrenic nerve palsy being a well-described complication.^4^^,^^5^ PFA may mitigate these risks owing to its myocardial selectivity, but vigilance remains essential.

Several potential pitfalls should be recognized by operators adopting this workflow. First, inadequate identification of the sinus node region may increase the risk of transient or persistent sinus node dysfunction; therefore, systematic activation mapping of the RA and SVC should be performed before ablation. Second, failure to adequately assess phrenic nerve capture can result in unintended nerve stimulation, underscoring the importance of high-output pacing before each application and voltage reduction when capture is observed. Third, improper balloon positioning—either too close to the sinus node or with insufficient circumferential wall contact—may increase the risk of bradyarrhythmic events or incomplete isolation.

1 patient developed a transient sinus node arrest immediately after the application, which resolved after short atrial pacing and isoprenaline infusion, suggesting reversibility of electroporation-related conduction block in this setting. After catheter repositioning, SVC isolation was successfully achieved without further complications.

No persistent conduction abnormalities or device-related adverse events occurred. These observations support the safety of the BiB-PFA system at the SVC but also emphasize that meticulous procedural planning, careful catheter positioning, and continuous monitoring are essential to avoid complications in this anatomically sensitive region.

### Clinical implications

Our findings support the feasibility of incorporating SVC isolation into ablation strategies for AF when using the BiB-PFA system. The reproducible workflow, short application time, and absence of major complications suggest that the BiB-PFA system may represent a safe and efficient approach for targeting this non-PV trigger. Given that phrenic nerve injury and sinus node dysfunction remain the primary concerns during SVC ablation, the ability of the BiB-PFA system to deliver energy with stable balloon contact and selective spline activation may provide procedural advantages. Larger studies and long-term follow-up are required to assess lesion durability and clinical impact on arrhythmia recurrence.

### Limitations

Several limitations should be acknowledged. First, the study was single center and included a small number of patients, limiting generalizability and statistical power. Second, follow-up was confined to the acute intraprocedural setting; no data on long-term outcomes or durability of SVC isolation are available. Third, the absence of a comparator arm prevents direct evaluation of potential advantages or disadvantages of the BiB-PFA system relative to other PFA platforms or conventional thermal energy.

## Conclusion

SVC isolation using the BiB-PFA system was feasible, fast, and safe, with acute success achieved in all patients and only 1 transient adverse event. These findings support the BiB-PFA system as a promising tool for adjunctive SVC ablation in patients with AF. Larger multicenter studies with long-term follow-up are warranted to confirm durability and define its role in clinical practice.

## Disclosures

R.R.T.: speaker (Pfizer, Abbott, Biosense Webster, Boston Scientific, Doctrina Med, cme4u, Medtronic, Radcliffe, Wikonect), consulting/advisory (Boston Scientific, Biosense Webster, Capvision, Guidepoint, Haemonetics, Medtronic, Philips, Abbott), institutional research (Biotronik, Abbott, Boston Scientific, Medtronic, LifeTech, Johnson & Johnson), travel (Biosense Webster, Abbott, Boston Scientific, Medtronic, Philips). C.E.: research/travel grants and speaker (Abbott, Boston Scientific, LifeTech, Biosense Webster, CardioFocus, C.T.I. GmbH, Doctrina Med). K.H.K.: grants/personal fees (Abbott Vascular, Medtronic, Biosense Webster; outside submitted work). S.H.: educational/travel grants (Abbott). J.P.W.: funding (German Foundation of Heart Research F/29/19), speaker (Abbott, Doctrina Med), travel (Boston Scientific).
